# Phytochemical Study of Eight Medicinal Plants of the Lamiaceae Family Traditionally Used as Tea in the Sharri Mountains Region of the Balkans

**DOI:** 10.1155/2020/4182064

**Published:** 2020-02-19

**Authors:** Avni Hajdari, Behxhet Mustafa, Lirie Hyseni, Ani Bajrami, Genista Mustafa, Cassandra L. Quave, Dashnor Nebija

**Affiliations:** ^1^Department of Biology, Faculty of Mathematical and Natural Science, University of Prishtina “Hasan Prishtina”, Mother Theresa St., 10000 Prishtina, Kosovo; ^2^Faculty of Natural Sciences, University of Tiran, Boulevard “Dëshmorët e Kombit”, Square “Mother Teresa” No. 183, 1001 Tirana, Albania; ^3^Faculty of Medicine, University of Prishtina “Hasan Prishtina”, St. Bulevardi I Dëshmorëve, 10000 Prishtina, Kosovo; ^4^Center for the Study of Human Health, Emory University, 1557 Dickey Drive, Room 306, Atlanta, GA 30322, USA; ^5^Department of Pharmaceutical Chemistry, Faculty of Medicine, University of Prishtina “Hasan Prishtina”, Bulevardi I Dëshmorëve, p.n. 10 000 Prishtina, Kosovo

## Abstract

In the present study, eight plant species belonging to Lamiaceae family were identified as ingredients for herbal teas in the region of Sharri Mountains: *Thymus serpyllum*, *Rosmarinus officinalis*, *Melissa officinalis, Origanum vulgare*, *Mentha longifolia*, *Ocimum basilicum*, *Teucrium chamaedrys*, and *Sideritis scardica,* respectively. Chemical composition of essential oils obtained from these species was analyzed using GC-MS and GC-FID with the aim of examining their volatile compound profiles, responsible for their respective flavors and fragrance. Principal Component Analysis (PCA) was performed with the aim of grouping plant species under study on the basis of their chemical composition. Experimental data revealed the typical volatile constituent pattern for the Lamiaceae family. Monoterpenes and sesquiterpenes, responsible for flavor and medicinal use of these plants, were the most abundant groups of the volatile constituents. PCA data analysis resulted in the grouping of these analyzed species in four principal clusters.

## 1. Introduction

The Sharri Mountains region (in Albanian known as Malet e Sharrit; in Macedonian and Serbian as Šar-Planina) is one of the biggest mountain massifs in Balkans (1,600 km^2^) and is situated in the Republic of North Macedonia (in a total area of 826.8 km^2^ or 51.44%) and Republic of Kosovo (in a total area of 780.4 km^2^ or 48.56%) [1]. This region serves as a tremendous reservoir of Traditional Environmental Knowledge (TEK) related to wild plants because of its complex biocultural diversity (hotspot of biodiversity and home to a variety of different ethnic and religious groups), the socioeconomic environment, and the long held traditions in the collection of wild plants by local inhabitants.

To date, there are roughly 1,500 vascular plant species known to grow in the Sharri Mountains [2, 3]. In addition to local species richness, the region is also rich in cultural, linguistic, and religious diversity. Various ethnic groups, including Albanians, Bosniaks, and Gorani (Muslims) and Serbs (Christian Orthodox) live on the Kosovo side of the Sharri, while the Macedonian side is inhabited by Albanians and Gorani (Muslims) and Macedonians (Christian Orthodox). These ethnic groups have lived in close contact over a span of many centuries in villages that are isolated from urban areas.

Local communities have benefited from the rich biodiversity of the region over centuries and for a variety of different purposes, including as ingredients in the creation of medicine, food, beverages, and snacks and as recreational teas. [2, 4–12]. Traditional uses of plants as recreational tea in Sharri have been reported in an ethnobotanical study, which covers other European countries as well [10]. In this article, we use the term “tea” to refer to herbal beverages prepared as infusions and that are consumed as food, excluding those teas prepared and consumed only for specific medicinal purposes, as well as black tea (*Camellia sinensis* (L.) Kuntze).

A review of the current literature revealed that study of the chemical composition of species found in Sharri was restricted primarily to those used mainly for pharmaceutical preparations [13–16]. Only a few reports on the chemical composition of species used for tea in the Sharri region and collected from Sharri have been published thus far. These include reports on the chemical composition of *Origanum vulgare* L. and *Malus sylvestris* Mill. collected from the Kosovo side of Sharri [17, 18] and another on the chemical composition of the essential oils of *Sideritis scardica* Griseb. from the North Macedonia side of Sharri [18].

Nevertheless, there is a growing interest in research on the chemical composition of specific herbal teas produced commercially in different regions of the world [19]. Hence, the aim of this study was twofold: first, to identify plant species which are traditionally used for herbal tea preparation in Kosovo through review of the ethnobotanical literature; second, to analyze their chemical composition and identify volatile compounds thought to be responsible for their flavor, fragrance, and health benefits.

## 2. Materials and Methods

### 2.1. Plant Material

Plants species were chosen based on the review of numerous ethnobotanical studies and published ethnographies, as well as unpublished fieldwork results originating from Sharri (Kosovo and North Macedonia). Species used in water infusions as aromatic and refreshing hot beverages (recreational tea) consumed in food and not for specific medicinal purposes were selected.

Plant material (specific plant tissues used to prepare tea) was either collected in the Sharri region from wild populations or purchased from the local markets, provided by local residents in Prizren (Kosovo) and Tetovo (Macedonia) that wildcrafted or cultivated the materials in Sharri for personal or family use. Plant material was collected from May to September and stored in a dry place protected from light until analyzed.

Taxonomic identification followed relevant standard botanical literature of the area [20]. Botanical nomenclature and family assignments followed the Plant List database [21], and voucher specimens were deposited at the University of Prishtina Herbarium.

### 2.2. Plant Material Extraction

Plant material was air-dried in the shade at room temperature, and volatile compounds were obtained by hydrodistillation, standard extraction method of volatile compounds, using a Clevenger apparatus for 2 hours. The extracts were stored at −18°C in a freezer until further analysis.

### 2.3. GC-MS Analysis

GC-MS analysis using an Agilent 7890A GC system coupled to a 5975C MSD (Agilent Technologies) was used for the separation and identification of essential oils constituents. The ionization energy was 70 eV. The separation was conducted in HP-5MS column (30 m × 0.25 mm i.d., film thickness 0.25 mm). The carrier gas used was He, with an initial flow rate of 0.6 mL/min and subsequently at a constant pressure of 50 psi. The carrier gas used was He with a constant flow rate 1.0 mL/min, injection temperature 280°C, injection volume 1.0 *μ*L, and a split ratio of 25:1. The initial GC oven temperature was 60°C (5 min) and was increased from 60°C to 200°C at a rate of 5°C/min.

### 2.4. GC-FID Analysis

GC-FID analyses were performed using an Agilent 7890A GC system equipped with an FID detector (Agilent Technologies). The separation was conducted on a HP-5MS column (30m × 0.1 mm, with 0.17 *μ*m film thickness). The carrier gas used was He, with an initial flow rate of 0.6 mL/min and subsequently at a constant pressure of 50°psi. The GC oven temperature increased from 60°C to 160°C at a rate of 10°C/min and subsequently to 280°C at a rate of 20°C/min., and the FID operated at 250°C with an air flow of 350 mL/min and a hydrogen flow of 35 mL/min. The injection volume was 1.0 mL.

### 2.5. Identification of Essential Oil Components

The identification of each of the components of the essential oil was performed by comparing their Kovats retention indices with those reported in the literature [22]. The Kovats index was calculated based on a linear interpolation of the retention times of a homologous series of *n*-alkanes (C9–C28) under the same operating conditions. The components were also identified by comparing the mass spectra of each constituent with those stored in the NIST 08 and WILEY MS 9^th^ databases and with mass spectra from the literature [22]. The percentage composition of the oils was computed using the normalization method from the GC peak areas, calculated as the mean of three samples, without correction factors.

### 2.6. Statistical Analysis

Principal Component Analysis (PCA) was used for multivariate association between volatile constituents and plant species in the study. Chemical compounds present in percentages higher than 3% were selected for statistical analysis. The XLSTAT program (version 2014.2.03) was used for the PCA and HCA.

## 3. Results and Discussion

In total, eight plant species belonging to the Lamiaceae family were identified as being used for making recreational herbal teas (Tables 1 and 2). Members of the Lamiaceae (and Rosaceae) family are well-known as local herbal teas in eastern and central Europe [10].

### 3.1. Statistical Analysis

Principal Component Analysis (PCA) has been used with the aim of grouping plant species of the Lamiaceae family, which have been used traditionally as teas on the basis of their chemical composition.

**Figure 1:**
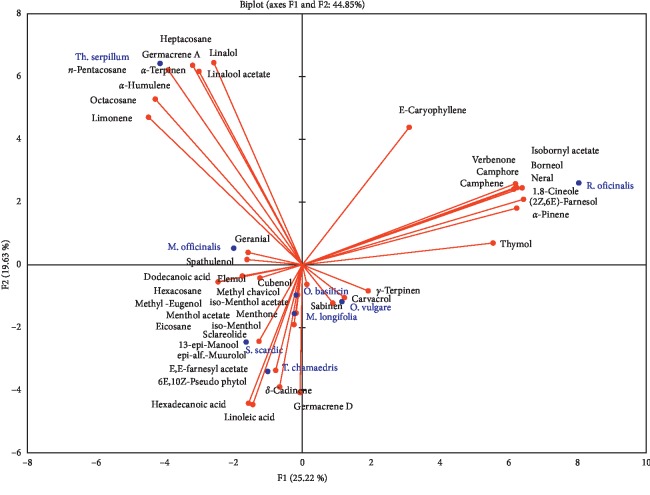
Diagram generated from the Principal Component Analysis of volatile compounds found in 8 plant species of the Lamiaceae family traditionally used as teas.

Principal Component Analysis demonstrated that eight plant species examined in this study have been grouped in four principal groups. *Thymus serpyllum* in the first group, *Rosmarinus officinalis* in the second group, and *Melissa officinalis* in the third group, whereas the fourth group included *Origanum vulgare*, *Mentha longifolia*, *Ocimum basilicum*, *Teucrium chamaedrys*, and *Sideritis scardica,* with greater similarities in relation to their chemical composition (Figure 1).

### 3.2. Chemical Composition of Volatile Compounds of Lamiaceae Species

Volatile compounds of eight species of the Lamiaceae family were analyzed in total, and results have been presented in Table 1.

#### 3.2.1. *Melissa officinalis* L., Lamiaceae

Twenty-three compounds were identified using GC-MS analysis of essential oils obtained from the distillation of aerial parts of the lemon balm*, Melissa officinalis* (Table 1). These compounds predominantly belonged to the class of oxygenated sesquiterpenes (28.56%) and oxygenated monoterpenes (20.38%), while percentage of sesquiterpenes was 9.18% followed by fatty acids and their derivatives, hydrocarbons, and monoterpenes with their respective percentages of 5.59%, 4.77%, and 3.78% (Table 1). The most abundant compounds were geranial (20.38%), spathulenol (18.61%), E-caryophyllene (7.32%), dodecanoic acid (5.59%), elemol (4.21%), limonene (2.61%), *n*-pentacosane (2.08%), and caryophyllene oxide (1.99%) (Table 1). In the lemon balm samples growing in Turkey, the monoterpene, D-limonene was the most prominent constituent (26.00%), citral and neral were more prominent oxygenated monoterpenes (14.93% and 13.6%, respectively), and caryophyllene oxide was the most abundant oxygenated sesquiterpene (11.45%) [23]. In samples from Greece, monoterpenes (*β*‐pinene and sabinene), sesquiterpene (E)-caryophyllene, and oxygenated sesquiterpene (caryophyllene oxide) were major constituents, and citral and citronellal were absent [24]. In Serbian commercial samples of the essential oil of lemon balm, the most prominent compounds identified in the essential oil were oxygenated monoterpenes, citronellal, geranial, *α*-terpineol, *α*-terpinyl acetate, neral, and the monoterpene, limonene. [25].

#### 3.2.2. *Mentha longifolia* L., Lamiaceae

In essential oils obtained from aerial parts of wild mint, *Mentha longifolia*, twenty-nine compounds were identified. The dominant components are oxygenated monoterpenes (89.46%) and monoterpenes (2.85%), followed by sesquiterpenes (5.43%) and oxygenated sesquiterpenes (2.05%). The most abundant compound is the oxygenated monoterpene, menthone (73.77%). In lesser amounts are present *iso*-menthyl acetate (4.1%), 1.8-cineole (3.69%), and isomenthol (3.35%) (Table 2). Peak areas of sesquiterpenes, *β*-ylangene and *γ*-muurolene, are 2.77% and 2.23%, respectively. In a report by Stanisavljević et al. [26], the main components of essential oils obtained from this species, dried by three different techniques, were piperitone, carvone, menthone, limonene, trans-caryophyllene, *γ*-muurolene, 1,8-cineole, and cis-dihydrocarvone. The principal constituents in the mint oil from Serbia were trans-dihydrocarvone (23.64%), piperitone (17.33%), and cis-dihydrocarvone (15.68%) [27], whereas in samples from Bosnia and Herzegovina, the main constituents of the essential oil of *M. longifolia* leaves were oxygenated monoterpenes, piperitone oxide (63.58%) and 1.8-cineole (12.03%), followed by the oxygenated sesquiterpene, caryophyllene oxide (4.33%), and sesquiterpenes, trans-caryophyllene (2.98%) and cis-caryophyllene (0.82%) [28]. Similarly, in samples from Croatia, the dominant constituents were oxygenated monoterpenes (69% of the oil), followed by monoterpenes and sesquiterpenes (31% of the oil) [29].

#### 3.2.3. *Ocimum basilicum* L., Lamiaceae

In essential oils obtained from the aerial parts of basil, *Ocimum basilicum*, fifty-six compounds were identified. Phenylpropanoids were the principal constituents, constituting 62.8%, followed by sesquiterpenes and their oxygenated derivatives (14.14 and 10.19% respectively). Oxygenated monoterpenes and monoterpene hydrocarbons were present in smaller amounts (11.55% and 1.3%, respectively). The phenylpropanoid derivatives, methyl chavicol (estragole) (45.82%) and methyl eugenol (12.1%), were the most prominent compounds. Sesquiterpenes and oxygenated sesquiterpenes (E-caryophyllene, 4.08%; elemol, 2.62%; spathulenol, 2.54%; bicyclogermacrene, 2.33%; *α-trans-*bergamotene, 1.97%; germacrene *D*, 1.81%) constituted the second abundant class of compounds (Table 1). The respective percentages of oxygenated monoterpenes, linalool and 1,8-cineole, were 4.77% and 3.89%, and the aromatic ketone, benzophenone, was present in the percentage of 2.7%. Therefore, experimental data of our study revealed that *O. basilicum* samples growing in Kosovo belonged to the *methyl chavicol* chemotype. The phenylpropanoid compound, methyl chavicol (estragole), was the main component from basil samples from Turkey (78.02%), followed by methyl eugenol (78.02%), *α*-cubebene (6.17%), nerol (0.83%), and *e*-muurolene (0.74%) [30]. In *O. basilicum* samples from Croatia, the major monoterpene alcohol, linalool (28.6%), was followed by estragole (21.7%). Other important compounds were (E)-methyl cinnamate (14.3%), alpha-cadinol (7.1%), eugenol (5.9%), 1,8-cineole (4.0%), methyl eugenol (3.1%), and *α-*bergamotene (2.2%) [31].

#### 3.2.4. *Origanum* vulgare L., Lamiaceae

Thirty-nine compounds were identified using GC-MS analysis of essential oils obtained from the distillation of aerial parts of the oregano*, Origanum vulgare* L. These compounds predominantly belonged to the class of sesquiterpenes and their oxygenated derivatives (36.92% and 10.57%, respectively) and monoterpenes and their oxygenated derivatives (23.32% and 18.26%, respectively). The most prominent compounds were germacrene *D* (14.2%), sabinene (11.16%), E-caryophyllene (14.9%), carvacrol (10.23%), cubenol (3.46%), *γ*-terpinene (3.23%), thymol (3.17%), bicyclogermacrene (2.88%), *d*-cadinene (2.87%), E-*β*-ocimene (2.72%), *β*-bisabolene (2.48%), Z-*β*-ocimene (2.18%), and caryophyllene oxide (2.18%) (Table 1).

In samples originating from Kosovo, essential oils were characterized by the presence of the following constituents: sabinene (1.81–12.34%), caryophyllene oxide (0.18–38.05%), 1,8 cineole (1.31–13.54%), *β*-caryophyllene with its isomers (E-) (0.48–14.0%) and (Z-) (4.63–12.33%), para-cymene (1.27–19.62%), *α*-terpineol (1.05–19.23%) and germacrene *D* (0.35–16.09%), *β*-ocimene with its isomers (Z-)-*β*-ocimene (0.76–7.6%) and (E)-*β*-ocimene (0.47–8.06%), spathulenol (0.41–5.39%), and linalool (0.99–4.37%). Regarding the composition of volatile compounds, the high percentages of oxygenated monoterpenes detected in our study (i.e., thymol and carvacrol) have also been reported in oregano sourced from Croatia, Italy, and Serbia [32–35].

#### 3.2.5. *Rosmarinus* officinalis L., Lamiaceae

Thirty compounds were identified in the essential oil obtained by hydrodistillation of the aerial parts of rosemary, *Rosmarinus officinalis*. The principal classes of compounds were oxygenated monoterpenes (53.47%) and monoterpenes (22.48%), followed by sesquiterpenes (14.15%) and oxygenated sesquiterpenes (9.58%). The most abundant oxygenated monoterpenes were 1.8-cineole (19.32%), camphor (13.71%), borneol (4.41%), isobornyl acetate (3.74%), thymol (3.1%), verbenone (3.00%), and *α*-terpineol (2.67%), whereas the principal monoterpenes were *α*-pinene (10.83%), camphene (3.28%), myrcene (2.54%), and *p*-cymene (2.58%). The percentage of sesquiterpene, E-caryophyllene, was 11.8%, while the peak areas of oxygenated sesquiterpenes khusinol and 7-epi-*α*-eudesmol were 2.37% and 2.29%, respectively. These results are in line with the published data on rosemary essential oil composition. Environmental, seasonal, and population-based variations in the chemical composition of rosemary essential oils have also been reported [36–39].

#### 3.2.6. *Sideritis scardica* Griseb., Lamiaceae

Sixty-four constituents were detected in the essential oils obtained from aerial parts of ironwort, mountain tea, *Sideritis scardica*. Fatty acids and hydrocarbons were the principal components (22.25% and 20.72%, respectively). Respective peak areas of oxygenated sesquiterpenes and sesquiterpenes were 14.74% and 12.92%. The less abundant constituents were oxygenated diterpenes (11.5%), diterpenes (4.31%), and monoterpenes (4.33%). The most prominent compounds were fatty acids, hexadecanoic acid (12.68%) and linoleic acid (3.99%); hydrocarbons, eicosane (10.89%), hexacosane (4.54%), and *n*-tricosane (2.31%); the oxygenated diterpene, 13-epi-manool (7.72%); oxygenated sesquiterpenes, sclareolide (5.52%) and caryophyllene oxide (2.09%); sesquiterpenes, E-caryophyllene (3.9%) and germacrene *D* (2.54%); the diterpene, isophyllocladene (2.33%); and monoterpene, *β*-pinene (2.35%). With regard to the composition of *S. scardica* essential oil, notable variability in its constituents has been previously reported. In samples originated from Sharri mountain (Ljuboten), essential oils were characterized by the presence of monoterpenes and sesquiterpenes (30.01% and 25.54%, respectively), and fatty acids and their esters and diterpenes (16.72–71.07% for fatty acids and their esters, and from 23.30 to 72.76%, for diterpenes) [40]. In Macedonian samples, the most prominent constituent was alpha-cadinol (20%), whereas *α*-pinene (4.4–25.1%) and *β*-pinene (2.8–18.0%) were predominant in the oil of Bulgarian samples. Greater levels of 1-octen-3-ol, phenylacetaldehyde, *β*-bisabolene, benzyl benzoate, and m-camphorene have also been noted [18].

#### 3.2.7. *Teucrium chamaedrys* L., Lamiaceae

Fifty compounds were identified in the essential oils obtained from the aerial parts of wall germander, *Teucrium chamaedrys*. Sesquiterpenes, hydrocarbons, and their oxygenated derivatives (49.3% and 11.3%, respectively), followed by fatty acids and their derivatives (23.9%), were the principal components of the essential oils. Monoterpenes and oxygenated monoterpenes made up 2.04% and 2.71% of the content, respectively, whereas the respective percentages of hydrocarbons and oxygenated diterpenes were 4.92% and 3.97%. The principal constituents were sesquiterpenes, germacrene *D* (24.1%); E-caryophyllene (3.96%); *β*-bourbonene (2.47%); *iso*-longifolene (2.2%); oxygenated sesquiterpene, caryophyllene oxide (2.09%); fatty acids, hexadecanoic acid (12.09%) and linoleic acid (6.03%); the oxygenated diterpene, pseudo-phytol (3.25%); the hydrocarbon, *n*-docosane (2, 94%); and unsaturated fatty alcohol, 1-octen-3-ol (2.92%). These results are in accordance with other reports in the literature. The main constituents of *T. chamaedrys* oils, *β*-caryophyllene (26.9%) and germacrene *D* (22.8%), have been reported by Kovacevic et al. [41] and the predominance of sesquiterpene hydrocarbons was observed in samples originating from Croatia [42].

#### 3.2.8. *Thymus serpyllum* L., Lamiaceae

Twenty-seven compounds were identified in the essential oils obtained from the distillation of aerial parts of wild thyme *Thymus serpyllum*. Oxygenated monoterpenes (44.81%) were the principal components, followed by sesquiterpenes (22.78%) and monoterpenes (14.17%). Hydrocarbons were present at 13.53%, whereas oxygenated sesquiterpenes and oxygenated diterpenes were present in smaller amounts (2.38% and 1.9%, respectively). The principal compounds were oxygenated monoterpenes, linalool (16.90%), linalool acetate (22.35%), and geranyl acetate (2.19%); sesquiterpenes, E-caryophyllene (8.43%), *α*-humulene (8.87%), and germacrene A (3.53%); and monoterpenes, *α*-terpinene (7.11%), limonene (3.10%), and E-*β*-ocimene (2.20%). Respective peak areas of hydrocarbon compounds, *n*-pentacosane, hexacosane, heptacosane, and octacosane, were 4.30%, 1.30%, 4.16%, and 2.94%. In *T. serpyllum* samples originating from Croatia and Bosnia and Herzegovina, monoterpenes were more abundant than oxygenated monoterpenes [43]. In Greece, GC-MS analysis of commercial samples revealed thymol as major component (38.50%), followed by *p*-cymene (8.9%), *γ*-terpinene (7.2%), bornyl acetate (7%), borneol (6%), and carvacrol (4.70%) [44]. On the other hand, in samples collected from two locations in Serbia, the principal components from the first locality (Mt. Kopaonik) were *trans*-caryophyllene (27.7%), *γ*-muurolene (10.5%), and *α*-humulene (7.5%), whereas in the second locality (Mt. Pasjaca) the principal components were trans-nerolidol (24.2%), germacrene *D* (16.0%), thymol (7.3%), *δ*-cadinene (3.7%), and *β*-bisabolene (3.3%) [45].

### 3.3. Frequency of the Species Used as Tea

Some of the plant species still have an important use as herbs in the cultures of the Sharri region.

Thus, the *Origanum vulgare* and *Thymus serpyllum* still are frequently used in regions, while *Sideritis scardica* is frequently used in Macedonia, as it is present in a larger population, while in Kosovo side of Sharri it is rarely used, as it grows in only a few small populations and this is an endangered species. *Melissa officinalis *, *Ocimum basilicum *, and *Mentha longifolia* are occasionally used to prepare tea. On the other hand, *Teucrium chamaedrys* and *Rosmarinus officinalis* are rarely used nowadays.

## 4. Conclusions

Over centuries, a considerable number of plant species in Sharri region have been used as traditional medicines, foods, and teas. In this study, eight plant species belonging to the Lamiaceae family were identified to be used as recreational tea based on review of the ethnobotanical literature. Chemical screening of the volatile constituents carried out with GC-MS and GC-FID revealed that most of these species have quite different chemical compositions (qualitatively and quantitatively) which contribute to the specific flavor and fragrance of these teas.

Further research is needed to address the preferences of the consumers on the tastes, fragrances, and colors of these recreational teas. Furthermore, research on the pharmacological, nutritional, and phytochemical properties of plants used for making tea is needed to ensure the safety and appropriateness of their use, especially for those consumed on a daily basis.

The wild plant species used for the preparation of tea represent an important national resource and therefore they need to be preserved and used in a sustainable way. The promotion of the traditional values of this group of plant species, besides their scientific importance, also represents their socioeconomic importance, since their promotion ecotourism, rural tourism, and other economic activities can be stimulated. This baseline data can be used for projects intended to foster rural development programs focusing on sustainable valorization of local herbal and wild food resources.

## Figures and Tables

**Table 1:** Phytochemical analysis of essential oils obtained from the extracts of plants used as teas, belonging to the Lamiaceae family.

Compound name and class	Kovats index	*Ocimum basilicum*	*Mentha longifolia*	*Teucrium chamaedrys*	*Sideritis scardica*	*Thymus serpyllum*	*Rosmarinus officinalis*	*Melissa officinalis*	*Origanum vulgare*
*α*-Pinene	939	0.25	0.46	1.34	1.33	—	10.83	—	0.49
Camphene	954	—	—	—	—	—	3.28	—	—
Sabinene	975	—	0.36	—	—	—	—	—	11.16
1-Octen-3-ol	979	—	—	2.92	—	—	—	—	—
*β*-Pinene	979	0.16	0.64	—	2.35	—	0.57	—	—
Myrcene	990	0.39	0.44	—	—	0.51	2.54	—	0.33
*o*-Cymene	1026	—	—	—	—	—	—	0.61	1.02
Limonene	1029	—	0.62	0.70	0.65	3.10	—	2.61	—
*p*-Cymene	1031	—	—	—	—	—	2.58	—	—
1.8-cineole	1031	3.89	3.69	—	—	—	19.32	—	1.44
Z-*β*-Ocimene	1037	—	0.17	—	—	1.25	—	—	2.18
E-*β*-Ocimene	1050	0.13	0.10	—	—	2.20	—	—	2.72
*γ*-Terpinene	1059	0.23	—	—	—	—	0.52	—	3.23
Linalool	1096	4.77	—	0.34	—	16.90	1.96	—	—
*α*-Terpinene	1017	—	—	—	—	7.11	—	—	—
Camphor	1146	—	1.37	—	—	—	13.71	—	—
Menthone	1152	—	73.77	—	—	—	—	—	—
Borneol	1169	0.20	—	—	—	—	4.41	—	0.49
Terpinen-4-ol	1177	—	—	—	—	—	1.02	—	1.45
Isomenthol	1182	—	3.30	—	—	—	-	—	—
*α*-Terpineol	1188	—	0.54	0.15	—	—	2.67	—	0.83
Dihydrocarveol	1193	—	—	—	—	1.70	—	—	—
Verbenone	1205	—	—	—	—	-	3.00	—	—
Linalool acetate	1254	—	—	—	—	22.35	—	—	—
Geranial	1267	—	—	0.80	—	—	—	20.38	—
Isobornyl acetate	1285	0.17	—	—	0.16	—	3.74	—	—
Thymol	1290	0.40	0.50	—	0.20	—	3.10	—	3.17
Carvacrol	1298	0.12	—	—	—	—	0.54	—	10.23
Menthyl acetate	1295	—	1.16	—	—	—	—	—	—
*iso*-Menthyl acetate	1304	—	4.10	—	—	—	—	—	—
*α*-Copaene	1376	0.14	—	0.66	0.65	—	—	—	—
E-methyl cinnamate	1378	1.05	—	—	—	—	—	—	—
Geranyl acetate	1381	—	—	—	—	2.19	—	—	—
*β*-Bourbonene	1388	—	—	2.47	0.28	—	—	—	0.82
*iso*-Longifolene	1390	—	—	2.20	—	—	—	—	—
*β*-Elemene	1390	0.57	—	—	1.02	—	—	—	0.29
Methyl eugenol	1403	12.10	—	—	—	—	—	—	-
E-Caryophyllene	1419	4.08	—	3.96	3.90	8.43	11.58	7.32	10.49
*Β*-Ylangene	1420	—	2.77	—	—	—	—	—	—
*α*-*trans*-Bergamotene	1434	1.97	—	—	—	—	—	—	—
*β*-Copaene	1432	—	—	1.04	—	—	—	—	0.23
α-Humulene	1454	0.14	0.13	0.87	—	8.87	0.39	0.22	1.47
E-*β*-Farnesene	1456	—	—	—	1.05	—	—	—	—
*γ*-Muurolene	1480	—	2.23	—	—	0.87	—	—	—
Germacrene D	1481	1.81	—	24.10	2.54	—	1.35	0.29	14.20
cis-Β-guaiene	1493	—	—	1.07	—	—	—	—	—
*β*-Macrocarpene	1499	—	—	1.81	—	—	—	—	—
Bicyclogermacrene	1500	2.33	0.18	—	—	1.08	—	0.30	2.88
*β*-Bisabolene	1505	—	—	0.95	—	—	0.45	—	2.48
Germacrene a	1509	—	—	—	—	3.53	—	—	—
Cubebol	1515	—	—	—	—	—	—	1.04	—
*δ*-Cadinene	1523	0.45	0.12	6.97	—	—	—	1.05	2.87
Zonarene	1529	—	—	—	1.30	—	0.38	—	—
*Γ*-Cuprenene	1533	0.47	—	1.62	—	—	—	—	—
Elemol	1549	2.62	—	—	—	—	—	4.21	1.39
Dodecanoic acid	1566	—	—	1.46	—	—	—	5.59	-
Spathulenol	1578	2.54	0.32	0.57	0.34	—	—	18.61	0.88
Caryophyllene oxide	1583	1.41	0.19	1.21	2.09	0.53	1.17	1.99	2.18
Benzophenone	1627	2.70	—	—	—	—	—	—	—
Caryophylla-4 (12), 8 (13)-diene-5*β*-ol	1640	—	—	—	0.25	—	1.87	—	—
Epi-α-muurolol	1642	—	—	1.00	—	—	—	—	1.63
Cubenol	1646	0.15	—	0.64	—	0.66	—	—	3.46
*α*-Cadinol	1654	0.33	1.10	0.70	1.45	0.54	—	—	—
7-Epi-α-eudesmol	1663	—	—	—	—	—	2.29	—	—
Khusinol	1676	—	—	—	—	—	2.37	—	—
Epi-*α*-bisabolol	1684	0.22	—	1.12	—	—	1.88	0.63	—
*α*-Costol	1774	—	—	—	1.24	—	—	—	—
Cyclopentadecanolide	1833	—	—	—	1.12	—	—	—	—
E,E-farnesyl acetate	1843	—	—	3.27	—	—	—	—	—
Phenyl ethyl octanoate.	1847	—	—	0.84	1.17	—	—	—	—
Isophyllocladene	1968	—	—	—	2.33	—	—	—	—
Hexadecanoic acid	1995	—	—	12.09	12.68	—	—	—	—
Eicosane	2000	—	—	—	10.89	—	—	—	—
Sclareolide	2066	—	—	—	5.52	—	—	—	—
N-Octadecanol	2077	—	—	—	1.08	—	—	—	—
Pseudo-phytol	2018	—	—	3.25	—	—	—	—	—
Linoleic acid	2133	—	—	6.03	3.99	—	—	—	—
Oleic acid	2142	—	—	—	1.83	—	—	—	—
*n*-Docosane	2200	—	—	2.94	0.75	—	—	—	—
*n-*Tricosane	2300	—	—	1.54	2.31	—	—	—	—
Isopimarol	2310	—	—	—	1.54	1.90	—	0.63	—
*n-*Pentacosan	2500	—	—	—	0.34	4.30	—	2.08	—
Hexacosane	2600	—	—	—	4.54	1.30	—	0.69	—
Heptacosane	2700	—	—	—	—	4.16	—	-	—
Octacosane	2800	—	—	—	1.18	2.94	—	1.74	—

Total		99.98	99.99	99.01	94.3	99.99	10−	72.89	89.07
Monoterpenes		1.30	2.85	2.04	4.33	14.17	22.48	3.78	23.32
Oxygenated monoterpenes		11.55	89.46	2.71	1.16	44.81	53.47	20.38	18.26
Sesquiterpenes		14.14	5.43	49.30	12.92	22.78	14.15	9.18	36.92
Oxygenated sesquiterpenes		10.19	2.05	11.03	14.74	2.38	9.58	28.56	10.57
Diterpenes		—	—	—	4.31	—	—	—	—
Oxygenated diterpenes		—	—	3.97	11.50	1.90	—	0.63	—
Fatty acids and derivatives		—	0.20	23.90	22.25	0.42	0.32	5.59	—
Hydrocbons		—	—	4.92	20.72	13.53	—	4.77	-
Phenylpropanoids and other compounds		62.8	—	0.75	0.50	—	—	—	—
Unknown		—	—	0.39	1,87	—	—	—	—

**Table 2:** List of the selected plant species used for tea preparation.

Plant species	Plant organs used	Plant origin	Frequency of use
*Origanum vulgare* L.	Aerial parts of plant	Wild	Still being used
*Thymus serpyllum* L.	Aerial parts of plant	Wild	Still being used
*Mentha longifolia* (L.) L.	Aerial parts of plant	Wild	Occasionally used
*Melissa officinalis* L.	Aerial parts of plant	Wild	Occasionally used
*Ocimum basilicum* L.	Aerial parts of plant	Cultivated	Occasionally used
*Teucrium chamaedrys* L.	Aerial parts of plant	Wild	Rarely used
*Sideritis scardica* Griseb.	Aerial parts of plant	Wild	Still being used
*Rosmarinus officinalis* L.	Aerial parts of plant	Cultivated	Rarely used

## Data Availability

No data were used to support this study.

## Abbreviations

GC-FID: Gas chromatography-flame ionization detection
GC-MS: Gas chromatography-mass spectrometry
HCA: Hierarchical cluster analysis
PCA: Principal Component Analysis.
